# 

**Published:** 1907-12-28

**Authors:** 


### CONTENTS.

| | PAGE |
|---|---|
| **The Advertisement of Quack Remedies** | 329 |
| **Soot and Smoke** | 330 |
| **Annotations—** | |
| Lord Kelvin—A Scale of Medical Fees—Evidence and the Professions | 331 |
| **Medical Opinion and Movement** | 332 |
| **Hospital Clinics—** | |
| The Failing Heart. By S. Russell Wells, M.D., B.Sc., M.R.C.P. | 333 |
| The Treatment of Acute Inflammation by Passive Congestion. Bier's Method. By Charles W. Cathcart, F.R.C.S. | 336 |
| **Points in Treatment—** | |
| The Reduction of Adipose Tissue by Fucus Vesiculosus | 339 |
| **Points in Diagnosis—** | |
| The Three Glass Test in Cases of Pyuria | 340 |
| **Points in Surgery—** | |
| Empyema | 341 |
| Some Diseases of the Male Genital System | 342 |
| **Diseases of Children—** | |
| Infantile Œdema of the Lower Limbs | 343 |
| **Practical Notes on Diagnosis and Treatment** | 344 |
| **The General Practitioner's Column—** | |
| Dermatitis Venenata. | 345 |
| **The Practitioner's Relaxations and Hobbies—** | |
| Meteorology as Study and Pastime | 346 |
| **Hospital Administration—** | |
| League of Mercy | 347 |
| Homes for Inebriates | 350 |
| The Hygienic Superiority of Gas Lighting | 350 |
| News and Coming Events | 351 |
| **Nursing Administration—** | |
| The Passing of 1907 | 352 |
| The Common Task | 353 |
| **Editor's Letter-Box—** | |
| Hæmoptysis and Nitrite of Amyl | 354 |

